# Supplementary figures and images for: Development of at-home sample collection logistics for large-scale seroprevalence studies
Source: PLoS One. 2021 Nov 4;16(11):e0258516. doi: 10.1371/journal.pone.0258516 (PMC8568136; doi:10.1371/journal.pone.0258516)

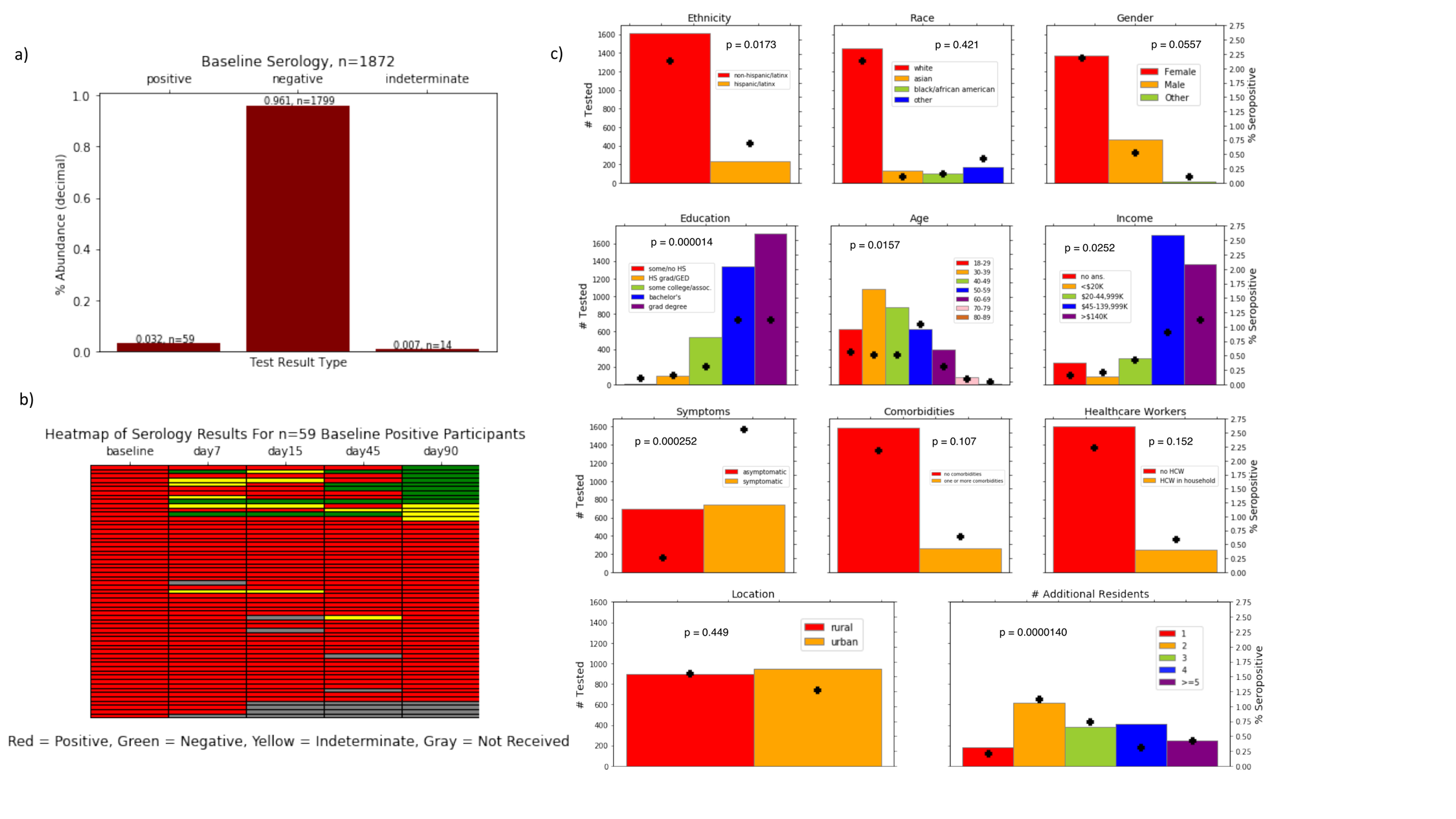

Supplement: S1 Fig — a) Distribution of positive, negative and indeterminate results for presence of total IgG antibodies against SARS-CoV-2 S1 protein across all individuals who returned a baseline test specimen (n = 1872). b) Heatmap showing presence of total IgG antibodies against SARS-CoV-2 S1 protein in follow-up samples of individuals who tested positive at baseline. Each row represents an individual and each column a time-point of sample collection (baseline, days 7, 15, 45 and 90) with data complete as of March 3, 2021. c) Histograms showing the total counts (left y-axis) for each variable in the study population. Black crosses represent percentage seropositivity (right y-axis) against the entire population (n = 1872) given individuals for each group. (TIF) [file pone.0258516.s003.tif]

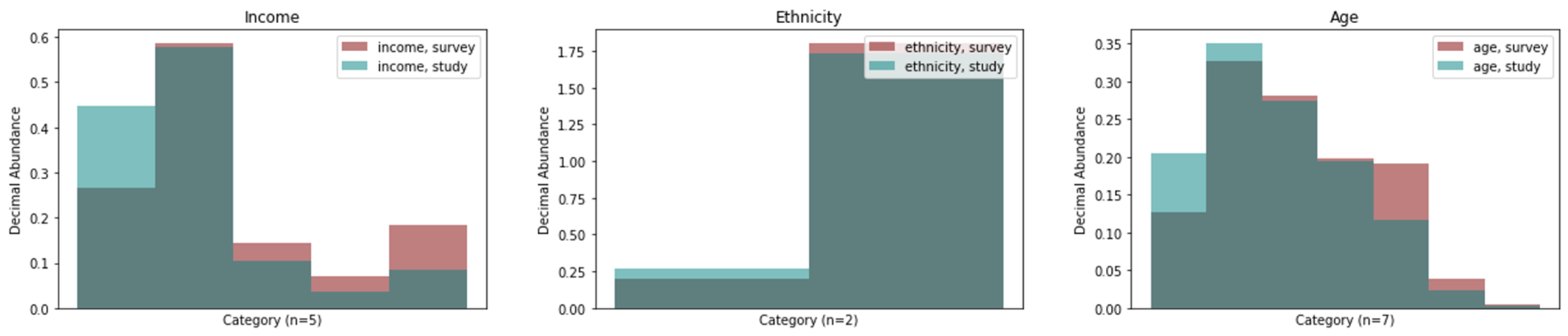

Supplement: S2 Fig — Comparison of general distribution of survey sample (maroon, n = 542) against general sample distribution of study population (green, n = 2066/as data is available for age, n = 2063) for the commonly collected demographic variables of income, ethnicity, and age. (TIF) [file pone.0258516.s004.tif]
